# Festival Dinners and Meetings

**Published:** 1893-07-22

**Authors:** 


### FESTIVAL DINNERS AND MEETINGS.

### THE COUNTESS OF DUFFERIN'S FUND.

The United Kingdom branch of this fund held its annual meeting, at Lord Brassey's, last week. Lady Dufferin was present and the chair was taken by Sir A. Lyall. It is now seven years since its first establishment, and in reviewing its work during that period the Chairman said that great progress had been made, and the movement had extended its organisation into nearly every province in India. Great difficulties in the way of political and social reform exist in India owing to the extraordinary diversity of the population and the number of provinces, but this particular charity was suited alike to all provinces and all races. It was the high-water mark of English civilisation in India, and all credit was due to Lady Dufferin, the founder. The annual report was presented by Sir J. Peile, who spoke of the wide range covered by the work of the association from Beloochistan to Rangoon, and from Lahore to Tinnevelly. More than 12 lakhs of rupees had been spent in erecting buildings specially intended to afford medical relief to native women, and the number of women who had benefited by medical aid during the past year was 466,000. Nine lady doctors with English qualifications, and 32 assistant surgeons and women doctors were working in connexion with the fund. The object of the United Kingdom branch is to raise subscriptions, bring the association before the public, and select lady doctors for the work. The association is entirely unsectarian. Speeches were also made by Sir A. Wilson and Surgeon-Captain A. W. Leahy.

### DENTAL HOSPITAL OF LONDON.

A conversazione, attended by a large number of guests, was given last week by the staff of the Dental Hospital at the galleries of the Royal Institute of Painters in Water Colours. Prizes were distributed during the evening by Professor Sir W. H. Flower, F.R.S., to students of the hospital school. Mr. Morton Smale, the Dean, stated that the school had a better record of success in the past year than ever before, the entries numbering 54, a dozen more than in any previous year. £40,000 was now urgently needed for the erection of a new hospital, towards which the sum of £9,500 had already been promised. Sir William Flower afterwards addressed the students, and the evening's entertainment concluded with a concert.

### LONDON HOMŒOPATHIC HOSPITAL.

The forty-third annual meeting was held at the hospital, 35, Queen Square, W.C., last week, Mr. J. Pakenham Stilwell presiding. The report showed that funds were urgently needed, it having been found necessary, not for the first time, to reduce the number of patients in order to avoid debt. It was feared that £10,000 more would be required for the building fund, but the board confidently hoped that the work being so far advanced it would not be allowed, by the many friends of the hospital, to stand still for lack of help. The meeting closed with the usual votes of thanks.

